# Glucocorticoids Elevate *Pseudomonas aeruginosa* Binding to Airway Epithelium by Upregulating Syndecan-1 Expression

**DOI:** 10.3389/fmicb.2021.725483

**Published:** 2021-11-01

**Authors:** Dong Liu, Ying-Ying Zeng, Meng-Meng Shi, Jie-Ming Qu

**Affiliations:** ^1^Department of Respiratory and Critical Care Medicine, Ruijin Hospital, Shanghai Jiao Tong University School of Medicine, Shanghai, China; ^2^Department of Respiratory and Critical Care Medicine, Huadong Hospital, Shanghai Medical College, Fudan University, Shanghai, China; ^3^Department of Respiratory and Critical Care Medicine, Zhongshan Hospital, Shanghai Medical College, Fudan University, Shanghai, China

**Keywords:** *Pseudomonas aeruginosa*, airway epithelium, bacteria binding, syndecan-1, glucocorticoids

## Abstract

Glucocorticoids are commonly used for the treatment of asthma and chronic obstructive pulmonary disease (COPD). Inhaled corticosteroids are associated with a significantly increased risk of pneumonia. Syndecan-1 (SDC1) located in the cell membrane of airway epithelial cell is the crucial molecule mediating infections by *P. aeruginosa* (PA). In the present study, we found that SDC1 expression was upregulated and the adhesion of PA to human bronchial epithelial (HBE) cells increased to 125 and 138%, respectively, after stimulation by dexamethasone or budesonide. The HBE cells knocking down SDC1 showed lower affinity to PA compared with control. CCAAT-enhancer-binding protein β (C/EBP β) and its phosphorylated form participated in the regulation of glucocorticoid to SDC1 for interfering C/EBP β or inhibiting phosphorylation of C/EBP β by LiCl and BIO, which are inhibitors of glycogen synthase kinase 3β (GSK-3β), and could prevent glucocorticoids from upregulating SDC1 expression. One should be cautious in administering glucocorticoids in chronic lung disease because of their property of increasing the expression of SDC1 and PA binding to the airway epithelium.

## Introduction

Administration of glucocorticoids (GCs) provides substantial clinical benefit to patients with asthma and chronic obstructive pulmonary disease (COPD). However, more and more clinical studies indicate that inhaled corticosteroids (ICS) were associated with a significantly increased risk of pneumonia (Singh et al., 2009). The risk was particularly elevated with high doses and shorter durations (Drummond et al., 2008). Another study comprised a large cohort of over 160,000 patients with COPD followed for a mean of 5 years with the conclusion that the risk of serious pneumonia was sustained with long-term use but declined after discontinuation. Both inhaled budesonide (BUDE) and fluticasone increased the risk of pneumonia with a more significant risk with fluticasone (Suissa et al., 2013). Administration of GCs is a predisposing factor of lung infection, but the exact mechanism is unclear. Stolberg and their colleagues carried out one animal study to target this question. Their research showed that ICS significantly increased the uptake of apoptotic cells by alveolar macrophages, which could inhibit pulmonary pneumococcal clearance (Stolberg et al., 2015). Although their identification offers a possible explanation for the molecular basis of this epidemiological association, the issue is far from settled.

Heparan sulfate proteoglycans (HSPGs) are glycoproteins, with the common characteristic of containing one or more covalently attached heparan sulfate (HS) chains, a type of glycosaminoglycan (GAG). There are three groups according to their location: membrane HSPGs, such as syndecans and glypicans, the secreted extracellular matrix HSPGs (agrin, perlecan, and type XVIII collagen), and the secretory vesicle proteoglycan, serglycin (Sarrazin et al., 2011). Park et al. (2001) reported 7-day-old syndecan-1^–/–^ (SDC1^–/–^) mice markedly resisted infection by intranasally inoculated *Pseudomonas aeruginosa* (PA). This result gives a strong proof to indicate that SDC1 is the crucial molecule mediating infection by PA, a clinically important Gram-negative bacterium in pneumonia and possibly in other infections in which the bacteria first encounter the host’s epithelia—a cell type whose predominant HSPG is SDC1. Peggy Benad-Mehner reported that dexamethasone (DEX) time- and dose-dependently increased SDC1 expression up to fourfold in breast cancer cell lines (Benad-Mehner et al., 2014). All these above studies provide adequate justification to develop the hypothesis that administration of GCs may influence the expression of SDC1 and its interaction with PA in airway epithelial cells.

In order to verify our hypothesis, we investigated the influence of DEX and BUDE to SDC1 expression *in vitro* and to the ability of binding PA to human airway epithelia cell line as well. The related molecular mechanism was further investigated. From the available data, we are unaware of any published reports involving the underlying molecular control of transcriptional interactions between GCs and SDC1. However, it was reported that CCAAT-enhancer-binding protein β (C/EBP β) was a key transcriptive factor regulating SDC1 expression (Larabee et al., 2011). C/EBP β mediated the effect of fluticasone propionate in host defense gene expression in BEAS-2B cells (Kimura et al., 2001; Zhang et al., 2007). Therefore, we explored whether GCs regulated SDC1 through transcription factor C/EBP β and its phosphorylation. Previous studies have proved that GSK-3β could interact with and phosphorylate C/EBP β (Lee et al., 2010). The phosphorylated C/EBP β by GSK-3β transactivated the subsequent gene expression (Tang et al., 2005). The rise in cellular cAMP augmented the activity of GSK-3β (Larabee et al., 2008). Thus, whether or not GCs could influence the cellular cAMP level in human bronchial epithelial (HBE) cells was also discussed in this study.

## Materials and Methods

### Airway Epithelial Cell Culture

The transformed 16-HBE cell line was kindly provided by Dr. Lin Shi of the central lab of Zhongshan Hospital, Fudan University and cultured at 37°C in RPMI (Hyclone) containing fetal calf serum 10% and antibiotics. To assess the effects of GCs, 90% confluent cells were exposed to DEX (Sigma-Aldrich) in various concentrations (10^–5^ M to 10^–7^ M). The concentration of BUDE (Sigma-Aldrich) was ranged from 2.3 × 10^–6^ M to 2.3 × 10^–9^ M (1 μg/ml to 1 ng/ml). To antagonize the effects of GCs, mifepristone (RU-486, Sigma-Aldrich) was used at a concentration of 10^–5^ M. 6MB-cAMP (Biolog Life Science Institute, Cat. No. M003) was used at a concentration of 10^–3^ M.

### Bacterial Strains

*Pseudomonas aeruginosa* strain O1 (PAO1) was obtained from the ATCC (ATCC 15692). GFP-labeled *P. aeruginosa* (GFP-PA) was kindly provided by Prof. Yuan-Lin Song, Zhongshan Hospital, Fudan University. Individual strain was routinely grown with shaking overnight in Luria-Bertani broth (LB broth) at 37°C, washed, and diluted to the appropriate concentration in Hanks’ balanced salt solution. Bacterial concentrations were determined by densitometry and confirmed by serial dilution followed by viable plate counts on appropriate agar media.

### Bacterial Binding Assay

*Pseudomonas aeruginosa* strain O1 or GFP-PA cells grown overnight in LB broth to stationary phase were diluted in 50 μl of serum-free RPMI and added to cells at a multiplicity of infection (MOI) of 20. After 1 h of infection at 37°C, adhesion assays were performed as described previously (Bucior et al., 2010). Briefly, cells were washed in PBS to remove non-adherent bacteria and were lysed in 1 ml of Ca^2 +^- and Mg^2 +^-free PBS with 0.25% Triton X-100 (Sigma-Aldrich) for 30 min. After lysis, cells were removed from the 96-well plates by gentle scraping. Bacteria were enumerated by plating serial dilutions of cell lysates on LB plates and counting the CFU.

### RNA Isolation and Real-Time PCR

RNA was isolated using RNAiso Plus (Takara Bio Inc., Dalian) according to the manufacturer’s protocol. Five hundred nanograms of RNA was reverse transcribed using PrimeScript^TM^ RT reagent Kit with gDNA Eraser (Takara Bio Inc., Dalian) and subsequently used for SYBR green-based real-time PCR using a standard protocol (Takara Bio Inc., Dalian). Primers (Sangon, Shanghai, China) used for gene expression: GAPDH (glyceraldehyde 3-phosphate-dehydrogenase) (NCBI GenBank: NM_002046): AGAAGGCTGGGGCTCATTG/AGGGGCCATCCACAGTCT TC; SDC1 (NCBI GenBank:NM_001006946): CTCTGGCT CTGGCTGTGC/GGTCTGCTGTGACAAGGTGA; C/EBP β (NCBI GenBank:NM_001285879.1): ACAGCGACGAGTA CAAGATCC/TGCTTGAACAAGTTCCGCAG.

PCR conditions were 95°C for 10 s followed by 40 cycles with 95°C for 5 s and 60°C for 34 s. The results were calculated applying the Δ ΔCT method and were presented in percentage increase relative to control.

### Protein Analysis

Cells were lysed with RIPA buffer (10 mM Tris–HCl, pH 7.4, 150 mM NaCl, 1 mM EDTA, 0.1% SDS, 1% Triton X-100, and 1% sodium deoxycholate) containing Complete Protease Inhibitor (Cell Signaling Technology) and PhosphoSTOP (Roche) for phosphoprotein detection. Protein concentration was determined by using the BCA Protein Assay Kit (Thermo Fisher Scientific), and equal amounts of proteins were separated by electrophoresis in 6% SDS-polyacrylamide gel for SDC1 and 12% gel for other proteins and resolved by electrophoresis (Mini Protean II apparatus; Bio-Rad) at 100 V in a buffer containing 2.5 mM Tris base (pH 8.3), 192 mM glycine, and 0.1% SDS. After electrophoresis, the proteins were transferred to a PVDF membrane (Millipore) with a 300-mA current for 90 min. Membranes were blocked by TBST with 5% milk for 1 h in room temperature. After blocking, the membranes were incubated in a 1:1,000 dilution of the primary antibody at 4°C overnight. The membranes were washed and then incubated in a 1:5,000 dilution of secondary horseradish peroxidase-conjugated goat (anti-rabbit/mouse) IgG (Santa Cruz) in blocking solution. Membranes were again washed and processed for detection of target protein by incubation in an appropriate substrate solution containing a luminol compound (SuperSignal Substrate; Thermo Fisher Scientific) for chemiluminescent signal development.

The following primary and secondary antibodies for western blot were used for detection of SDC1 (AbD Serotec), glucocorticoid-receptor (GR; Cell Signal Technology), C/EBP β (Abcom), Phospho-C/EBP β (Cell Signal Technology), and Glycogen synthase kinase 3β (GSK-3β; Cell Signal Technology).

### Cell Surface Syndecan-1 Was Detected by Immunofluorescence

Cells were seeded into 12-well Culture Slide at 3 × 10^4^ cells per well and fixed in 100% cold methanol on ice for 10 min followed by 4% paraformaldehyde at room temperature for 2 min. Cells were blocked with 2.5% goat serum and 2% BSA in TBS for 1 h at room temperature, then incubated with 1:400 primary antibodies for SDC1 (AbD Serotec) in 2% BSA in TBST overnight at 4°C. Secondary antibodies (Alexa Fluor 488 goat anti-mouse IgG or Alexa Fluor 568 or 594 goat anti-mouse IgG, 1:500, Thermo Fisher Scientific) were applied in 2% BSA in TBST for 1 h at room temperature. Cell nucleus was stained with DAPI for 5 min. Slides were cover-slipped using Fluoromount-G (eBioscience) and images were captured with inverted microscope (Leica Microsystems).

### Confocal Microscope Images for Adhesion of *Pseudomonas aeruginosa* to Human Bronchial Epithelial

Human bronchial epithelial cells were seeded into 12-well Culture Slide at 3 × 10^4^ cells per well. GFP-PA grown overnight in LB broth to stationary phase were diluted in 50 μl of serum-free RPMI and added to cells at a MOI of 20:1. After 1 h of infection at 37°C, cells were washed in PBS to remove non-adherent bacteria and then fixed in 100% cold methanol on ice for 10 min followed by 4% paraformaldehyde at room temperature for 2 min. The following steps are the same as the immunofluorescence detection. Images were captured with confocal microscope (Zeiss LSM 800).

### siRNA Transfection

siRNA targeting SDC1 or C/EBP β was transfected into HBE cells with the transfection reagent TianFect (Tiangen) using the manufacturer’s protocol. Briefly, 1.2 × 10^5^ cells were plated into each well of a 24-well plate. Twelve hours after plating the cells, the transfection mixture containing 6 μl of TianFect and 100 nmol siRNA was prepared in 50 μl of Opti-MEM (Thermo Fisher Scientific). After 10 min of incubation, the transfection mixture was added to the cells, bringing the volume to 400 μl. After a 6-h exposure, the medium was removed and replaced with fresh RPMI containing 10% FBS. The sequence of the siRNAs against SDC1 was ggagacagcatcagggtta. The siRNAs against C/EBP β (Cat. stB0005928C-1-5, Ribobio, Guangzhou, China) contains a pool of three to four target-specific siRNAs. As a negative control, cells were transfected with a scrambled sequence not targeting any known gene.

### Lentivirus Packaging and Stable Transfection Cell Line Generation

Lentiviral constructs were designed by the HANYINBT (Shanghai). The HBE cells were stably transfected with hU6-MCS-CMV-ZsGreen 1-PGK-Puro negative control vectors and lentivirus (SH-SDC1) for knocking down SDC1. Target cells (1 × 10^5^) were transfected at a lentivirus/medium ratio of 1:50 in the presence of 5 μg/ml polybrene. Puromycin (2 μg/ml) was used to screen the stable transfected HBE cells (SH-HBE). Real-time qPCR and immunofluorescence were used to assess the efficiency of knocking down SDC1 expression in HBE.

### Cellular cAMP Measuring

Cellular cAMP was measured as the manufacturer’s protocol (Neweast Bioscience, Cat. No. 80203). Briefly, 3 × 10^4^ cells in 100 μl of RPMI with 10% FBS were plated into each well of a 96-well plate. When the confluence reaches about 90%, HBE cells were treated with DEX (10^–5^ M) or BUDE (2.3 × 10^–6^ M, 1 μg/ml) for 1 h in the presence or absence of RU-486 (10^–5^ M) simultaneously. Cellular cAMP was measured by ELISA method.

### Co-immunoprecipitation

Co-immunoprecipitation was performed with a nuclear extraction kit (Active Motif). The nuclear extracts containing 200 μg of proteins were incubated overnight at 4°C with 2 μg of anti-GR antibody (Cell Signal Technology) in 500 μl of NP-40 lysis buffer. Protein A/G beads (60 μl, Thermo Scientific) were added to the mixture and incubated for an additional hour with rocking. The immunocomplexes were then washed six times with NP-40 lysis buffer. The immunoprecipitated proteins were dissolved in 60 μl of 2× Laemmli buffer and boiled for 5 min before analysis by western blotting.

### Statistical Analysis

Data are expressed as means ± SD (standard deviation). Statistical significance was estimated by Student’s *t*-test using SPSS (19.0 version). Differences were considered to be significant at *p* < 0.05.

## Results

### Expression of Syndecan-1 Gene Was Influenced by Glucocorticoids

**FIGURE 1:**
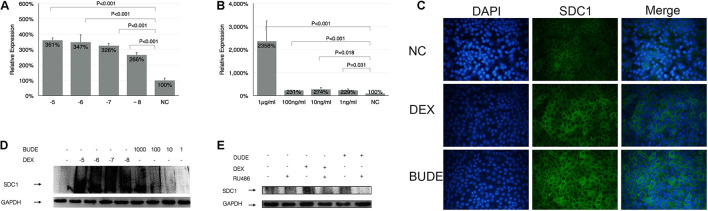
Glucocorticoid enhanced the expression of SDC1. DEX or BUDE increased SDC1 expression by qPCR **(A,B)**, by immunofluorescence staining **(C)**, and by western blot **(D)**. SDC1 is displayed in green (Alexa Fluor 488) and the nucleus is shown in blue (DAPI) in **(C)**. The receptor antagonist RU-486 inhibited SDC1 expression triggered by DEX or BUDE by western blot **(E)**. Student’s *t*-test was used to analyze the different groups. Six replications and three independent experiments. Magnification: 400× **(C)**.

As shown by Figures 1A,B, SDC1 expression was dose dependently induced 4 h later by GCs administration *in vitro*. Both DEX and BUDE increased SDC1 expression with a large concentration span from 10^–5^ M to 10^–7^ M of DEX and 2.3 × 10^–6^ M to 2.3 × 10^–9^ M (1 μg/ml to 1 ng/ml) of BUDE. The expression even soared more than 23-fold compared to those without stimulation of BUDE. Immunofluorescence staining (Figure 1C) and western blot (Figure 1D) verified the results from real-time quantitative PCR. SDC1 was mainly located at the HBE cell surface. SDC1 expression was observed in control HBE cells with a significant increase after 24 h stimulation by DEX (10^–5^ M) or BUDE (2.3 × 10^–6^, 1 μg/ml) (Figure 1C). The DEX- or BUDE-dependent induction of SDC1 was inhibited by the GR antagonist RU-486 (10^–5^ M, incubation for 24 h) (Figure 1E). From Figure 1, we could see that SDC1 expression was increased by glucocorticoid treatment, which was GR dependent.

### Upregulated Syndecan-1 Increased Adhesion of *Pseudomonas aeruginosa* to Human Bronchial Epithelial

**FIGURE 2:**
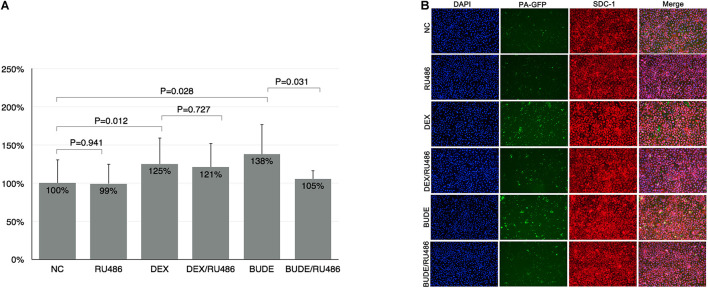
Glucocorticoids enhanced the adhesion of PA to HBE. DEX or BUDE increased the adhesion of PA to HBE assessed by CFU counting **(A)** and by confocal microscopy experiment **(B)**. RU486 inhibited the effects of glucocorticoids. SDC1 was stained by red (Alexa Fluor 568). PA was labeled by GFP (green). The nucleus was stained by DAPI (blue). Student’s *t*-test was used to analyze the different groups. Six replications and three independent experiments. Magnification: 400× **(B)**.

The binding experiments were carried out to show the relationship between the SDC1 expression level and the adhesion of PA to HBE. As shown by Figure 2A, the adhesion of PA to HBE increased to 125 and 138%, respectively, after stimulation by DEX (10^–5^ M) or BUDE (2.3 × 10^–6^, 1 μg/ml) assessed by bacterial binding assay *in vitro* (CFU counting). RU-486 (10^–5^ M) could reverse this function of GCs partially. The confocal microscope was used to show the affinity of PA to HBE cells. From Figure 2B, DEX or BUDE increased the fluorescence intensity significantly (red for SDC1). At the same time, the adhesion of PA was upregulated by DEX or BUDE compared with normal control group. RU486, the inhibitor of GR, reversed these functions.

### Knocking Down of Syndecan-1 Decreased the *Pseudomonas aeruginosa* Adhesion to Human Bronchial Epithelial

**FIGURE 3:**
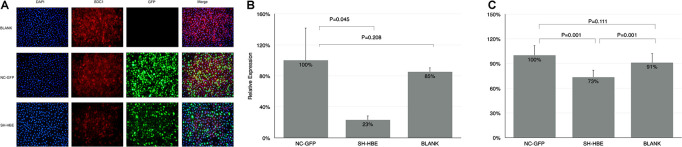
The adhesion of PA was reduced in the SDC1 knockdown HBE. **(A)** SDC1 was successfully downregulated by lentivirus vector carrying SH-RNA (SDC1-SH) assessed by immunofluence method. SDC1 was stained by red (Alexa Fluor 568). Both NC vector and SDC1-SH vector were labeled by GFP (green). The nucleus was stained by DAPI (blue). The qPCR was used to verify the knocking down of SDC1 **(B)**. The adhesion of PA to the knocking down SDC1 HBE cells assessed by CFU counting was decreased compared with that to control HBE cells **(C)**. Student’s *t*-test was used to analyze the different groups. Eight replications and three independent experiments. Magnification: 400× **(A)**.

In order to study whether the adhesion level of PA to HBE is associated with the expression of SDC1, the HBE cells knocking down SDC1 were established by lentivirus (SH-HBE). When we established the lentiviral-mediated knockdown of SDC1 in HBE cells, three sequences were used to select the most effective one (data not shown). From Figure 3A, the expression of SDC1 stained by red fluorescence was downregulated in the cells transfected by SDC1-SH vectors compared with that of NC-GFP and Blank groups. In Figure 3B, quantitative real-time PCR results showed that the SDC1 was downregulated to 23% in SH-HBE compared with that in NC-HBE. The adhesion of PA was decreased to 73% (Figure 3C) in the SH-HBE in which SDC1 was downregulated to 23% (Figure 3B). These results showed that the adhesion of PA to HBE was positively correlated with the expression level of SDC1.

### Syndecan-1 Expression by Glucocorticoids Associated With CCAAT-Enhancer-Binding Protein β

**FIGURE 4:**
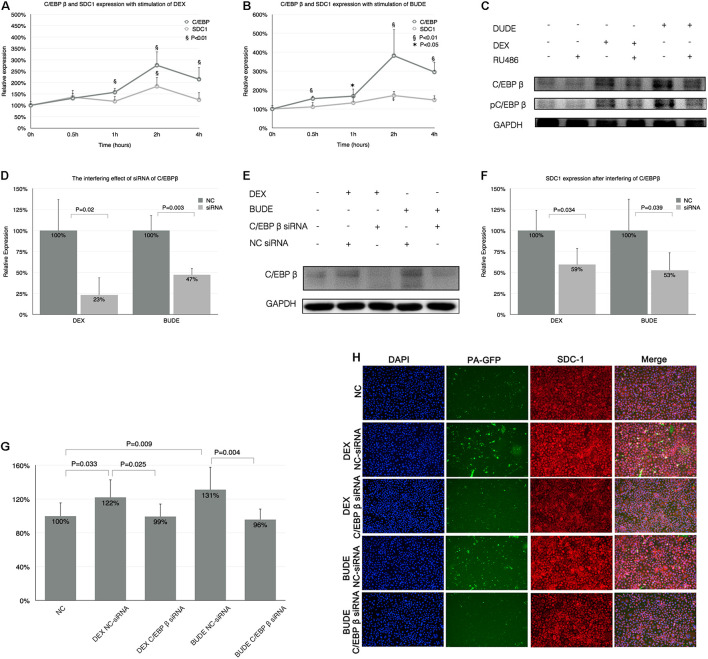
Syndecan-1 expression by GCs associated with C/EBP β. **(A,B)** The time course of SDC1 and C/EBP β expression after stimulation of DEX (10^–5^ M) or BUDE (2.3 × 10^–6^ M, 1 μg/ml) by qPCR. “*” indicates *p* < 0.05, “§” indicates *p* < 0.01. **(C)** C/EBP β protein and its phosphorylation increased with exposure of GCs and RU-486 (10^–5^ M) reversed these processes analyzed by western blot. C/EBP β was downregulated by C/EBP β siRNA in HBE cells with incubation of DEX or BUDE assessed by qPCR **(D)** and western blot **(E)**. Silencing C/EBP β decreased SDC1 expression assessed by qPCR **(F)** and reversed augmented adhesion of PA stimulated by GCs assessed by CFU counting **(G)** and confocal microscopy **(H)**. SDC1 was displayed in red (Alexa Fluor 594) and the nucleus in blue (DAPI). Magnification: 400× **(H)**. Student’s *t*-test was used to analyze the different groups. Four replications for qPCR, eight replications for CFU counting and three independent experiments.

It was reported that C/EBP β was a key transcriptive factor regulating SDC1 expression. In this section, we discussed whether C/EBP β influenced the SDC1 expression. Figures 4A,B show the time course of SDC1 and C/EBP β expression stimulated by DEX or BUDE. The expression patterns of SDC1 and C/EBP β were very consistent. C/EBP β expression was earlier than SDC1 for it increased statistically significantly at half an hour (BUDE, 2.3 × 10^–6^, 1 μg/ml) or 1 h (DEX, 10^–5^ M) after the stimulation of GCs while SDC1 did not.

CCAAT-Enhancer-Binding Protein β protein expression and its phosphorylation increased after GC stimulation (Figure 4C) and this process was GR dependent for RU486 (10^–5^ M)-inhibited C/EBP β expression and its phosphorylation.

We designed three siRNAs for interfering C/EBP β and the most efficient one could decrease C/EBP β to 23 or 47% with the exposure of DEX (10^–5^ M) or BUDE (2.3 × 10^–6^, 1 μg/ml) by qPCR (Figure 4D) or western blot (Figure 4E). Figure 4F shows that the increase of SDC1 with GC exposure was inhibited by C/EBP β siRNA analyzed by qPCR. When the C/EBP β was downregulated by siRNA, the increased adhesion of PA stimulated by GCs decreased compared with that in the group of NC-siRNA for C/EBP β assessed by CFU counting (Figure 4G) and confocal microscopy (Figure 4H).

### CCAAT-Enhancer-Binding Protein β Phosphorylation Was Dependent on Glycogen Synthase Kinase 3β

**FIGURE 5:**
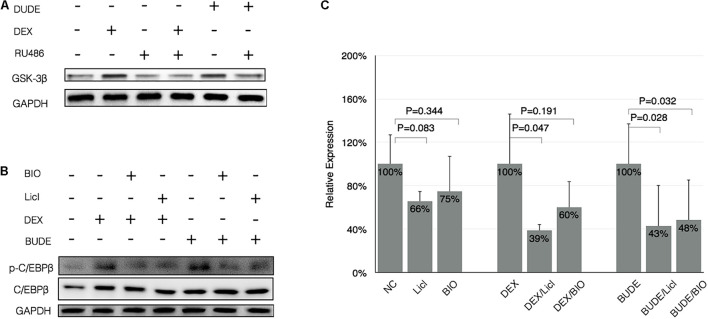
CCAAT-enhancer-binding protein β phosphorylation was dependent on GSK-3β. **(A)** DEX (10^–5^ M) or BUDE (2.3 × 10^–6^ M, 1 μg/ml) exposure increased expression of GSK-3β assessed by western blot, and the addition of RU-486 reduced levels of GSK-3β in GCs-exposed HBE. **(B)** GSK-3 inhibitors (20 mM LiCl or 5 μM BIO) reduced the GCs-induced phosphorylation of C/EBP β. **(C)** GSK-3 inhibitors reduced the expression of SDC1 with or without GC incubation assessed by real-time qPCR. Student’s *t*-test was used to analyze the different groups. Four replications for qPCR and three independent experiments.

Glycogen synthase kinase 3β (GSK-3β) is a serine-threonine kinase in the classic Wnt cell signal. GSK-3β was demonstrated to participate into the C/EBP β phosphorylation in a previous study (Lee et al., 2010). The phosphorylated C/EBP β by GSK-3β acquired DNA-binding function and transactivated the subsequent gene expression (Tang et al., 2005). We hypothesized that GSK-3β may be involved in the phosphorylation of C/EBP β at Thr-235 in GC-exposed HBE. To examine this possibility, the ability of GCs to induce C/EBP β phosphorylation was examined in HBE exposed to GSK-3 inhibitors. In Figure 5A, GC exposures lead to increased levels of GSK-3β, and RU486 reduced GSK-3β expression with GCs. In Figure 5B, the GC-induced phosphorylation of C/EBP β at Thr-235 was reduced by two different GSK-3 inhibitors, LiCl and BIO. These data suggest that GSK-3β was critical for the phosphorylation of C/EBP β at Thr-235 during GC stimulation (Figure 5C). The RT-qPCR results indicated that SDC1 was decreased in HBE cells with GSK-3 inhibitors, LiCl and BIO, which further suggests that inhibiting phosphorylation of C/EBP β by controlling of kinase activity of GSK-3β downregulated expression of SDC1, the critical binding protein of PA. Consequently, it is reasonable to speculate that the inhibition of SDC1 expression during the stimulation of GCs by GSK-3β inhibitors can reduce the adhesion of PA to respiratory epithelial cells.

**FIGURE 6:**
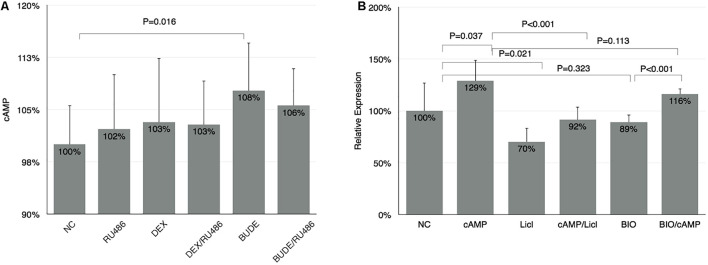
The non-genomic effect of GCs on SDC1 expression. **(A)** BUDE (2.3 × 10^–6^ M, 1 μg/ml) incubation increased cellular cAMP. RU-486 (10^–5^ M) did not reverse the effect of BUDE. Cellular cAMP was measured by ELISA method. **(B)** 6MB-cAMP triggered SDC1 expression up compared with control analyzed by qPCR. Student’s *t*-test was used to analyze the different groups. Four replications for qPCR and ELISA and three independent experiments.

Glucocorticoids upregulates SDC1 expression mainly by genomic signaling. However, RU486 did not reverse the function of BUDE or DEX completely (Figure 1E), which may imply the non-genomic effects of glucocorticoids. It was reported that the rise in cellular cAMP augmented the activity of GSK-3β by reducing the phosphorylation at Ser-9 (Larabee et al., 2008). Thus, we planned to detect whether or not GCs could increase the cellular cAMP in HBE cells. As shown in Figure 6A, BUDE (2.3 × 10^–6^ M, 1 μg/ml) incubation for 1 h increased cellular cAMP, which was not RU486 dependent. The cells treated by DEX (10^–5^ M) trended to increase but did not reach statistical significance. SDC1 expression triggered by 6MB-cAMP increased, but not as significant as GC incubation, which indicated that the genomic effect of GCs was the major factor triggering SDC1 expression. When the HBE cell was treated with a membrane-permeable analog of cAMP (6MB-cAMP), SDC1 expression increased to 129% compared with control (Figure 6B). GSK-3β inhibitors reversed the rising function of 6MB-cAMP to SDC1 in HBE partly. GSK-3β was important for maintaining SDC1 expression at a physiological level in HBE for that the GSK-3β inhibitor (LiCl or BIO) alone downregulated SDC1 expression (Figure 6B). GSK-3β inhibitor exposure reversing the function of 6MB-cAMP to SDC1 further verified that cAMP could activate GSK-3β and stimulate subsequent target gene expression. The above results show that although cAMP did not play a major role in the regulation of SDC1 expression, it still contributed to the process to a certain extent, which affected the affinity of PA to epithelial cells.

### Glucocorticoid Combined With CCAAT-Enhancer-Binding Protein β Regulated Expression of Syndecan-1

**FIGURE 7:**
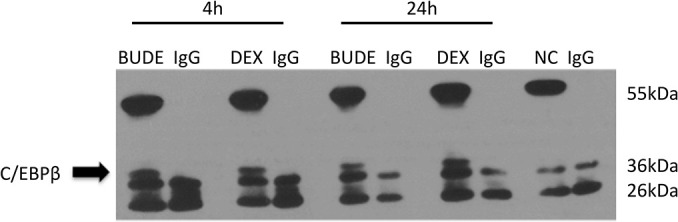
Glucocorticoid receptor combined with C/EBP β regulated expression of SDC1. HBE cells were exposed to Dex (10^–5^ M) or BUDE (2.3 × 10^–6^ M, 1 μg/ml) for 4 h or 24 h. In the immunoprecipitates of GR, C/EBP β was detectable in GC-treated cells but not in control cells.

The co-immunoprecipitation experiment was performed to examine whether GR interacted with C/EBP β during its transactivation of SDC1. HBE cells were exposed to DEX (10^–5^ M) or BUDE (2.3 × 10^–6^ M, 1 μg/ml) for 4 or 24 h. In the immunoprecipitates of GR, C/EBP β was detectable in GC-treated cells but not in vehicle-treated cells (Figure 7). This result indicates the interaction of the GR and C/EBP β for GCs action.

## Discussion

The adhesion of PA to HBE increased significantly after GC stimulation assessed by bacterial binding assay and confocal microscopy images. C/EBP β and its phosphorylated form participated in the regulation of glucocorticoid to SDC1 for interfering C/EBP β or inhibiting phosphorylation of C/EBP β by LiCl and BIO, which are inhibitors of GSK-3β, and prevented glucocorticoids from upregulating SDC1 expression. Our study may give a clue to explain why using glucocorticoids are associated with a significantly increased risk of pneumonia. To our knowledge, the present study is the first one to target this issue from a bacterial binding aspect.

Epithelial cells are lined on the mucosal surfaces of respiratory tracts and serve as the primary barrier against the entry of most infectious agents. PA is a ubiquitous opportunistic pathogen of humans and it chronically colonizes the lungs of patients with cystic fibrosis (CF) or other chronic lung diseases, leading to severe pulmonary damage and death.

Syndecan-1 is mainly expressed in placenta and bronchial epithelial cells (Su et al., 2004). SDC1 was reported to mediate PA binding to the airway epithelium through a flagella- and pili-dependent (Bucior et al., 2012) and –independent manner (Plotkowski et al., 2001; Imberty et al., 2004; Chemani et al., 2009; Bucior et al., 2010). SDC1 is preferentially expressed on the basolateral surface of polarized epithelium (Bishop et al., 2007). Chronic inflammation leads to epithelial injury and the exposure of the basolateral surface to the bacteria. Our results proved that GC administration increased the expression of SDC1 significantly. The upregulated SDC1 lead increased PA adhesion to HBE. Knockdown of SDC1 in HBE showed low affinity to PA, which highlights that SDC1 plays a critical role during PA adhesion. SDC1 is also known to mediate binding of various bacterial and viral pathogens besides PA (Freissler et al., 2000; Surviladze et al., 2012). Freissler et al. (2000) showed that overexpression of SDC1 and SDC4 leads to a three- and sevenfold increase in *Neisseria gonorrhoeae* invasion, respectively. *Streptococcus bovis*, *Streptococcus agalactiae*, *Streptococcus pyogenes*, *Staphylococcus aureus*, and *Staphylococcus epidermidis* showed increased adhesion to transfected ARH-77 cells with high expression of SDC1 (Henry-Stanley et al., 2005). Accordingly, it can be speculated that its increase has specific importance in pneumonia after giving GCs.

Glucocorticoid treatment causes robust changes in gene expression. We are unaware of any published reports involving the underlying molecular control of transcriptional interactions between GCs and SDC1. Our results showed that the upregulation of SDC1 was GR dependent for RU486, the inhibitor of GR, and reversed SDC1 increase by GCs.

**FIGURE 8:**
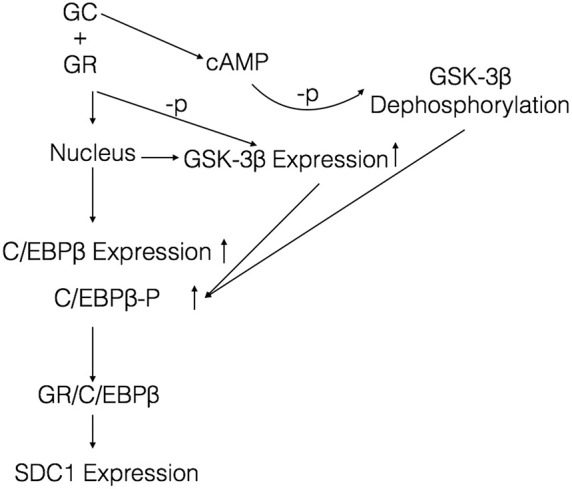
Proposed model depicting a signaling mechanism of GCs to activate SDC1 expression in HBE. Once GCs are delivered to the cytoplasm, GCs bind to GR and enter the nucleus to active the expression of C/EBP β and GSK-3β. GCs also increase the generation of cellular cAMP, which augments the activity of GSK-3β by reducing the phosphorylation. The increased GSK-3β enhances phosphorylation of C/EBP β. The activated C/EBP β binds to GR and induces SDC1 expression.

Glucocorticoid modulates transcription of a few target genes in a unique way that requires the receptor to dimerize with itself or other transcription factors. It was reported that C/EBP β was a key transcriptive factor regulating SDC1 expression (Larabee et al., 2011). Therefore, we explored the mechanism whether GCs regulate SDC1 through the transcription factor C/EBP β. From our results, we found that GCs upregulated the expression of C/EBP β in a time-dependent way. The increase of C/EBP β was earlier than SDC1. Knockdown of C/EBP β by siRNA significantly blocked the expression of SDC1 triggered by GCs. Phosphorylation of C/EBP β at Thr-235 by GSK-3β was also involved in the SDC1 expression in GC-exposed HBE. These results support the notion that C/EBP β and its phosphorylation level promote the transcription of SDC1 regulated by GCs. Our data are consistent with the literature showing that C/EBP β mediates the effect of fluticasone propionate in host defense gene expression in BEAS-2B cells (Kimura et al., 2001; Zhang et al., 2007). GSK-3β is a serine-threonine kinase in the classic Wnt cell signal. Previous studies have proved that GSK-3β can interact with and phosphorylate C/EBP β, supporting the present results (Lee et al., 2010). The phosphorylated C/EBP β by GSK-3β acquired DNA-binding function and transactivated the subsequent gene expression (Tang et al., 2005). Immunoprecipitate results showed that GR combined with C/EBP β regulated expression of SDC1 (Figure 7). Taken together, these results suggest a model where GCs trigger expression of GSK-3β and C/EBP β, leading the phosphorylation of C/EBP β on Thr-235 by GSK-3β and enhancing the ability of C/EBP β to combine GR and activate SDC1 transcription (Figure 8).

Our results verified that GCs increased cellular cAMP slightly and SDC1 expression increase was triggered by cAMP mimics (6MB-cAMP). cAMP regulated the expression of SDC1 by GSK-3β in that GSK-3β inhibitors’ exposure reversed the function of 6MB-cAMP to SDC1, which further verified that cAMP could activate GSK-3β and stimulate subsequent target gene expression. Kaur et al. (2008) reported that cAMP-elevating agents and a cAMP mimetic could enhance simple GRE-dependent transcription *via* the classical cAMP–PKA pathway. Further studies are needed to demonstrate whether or not the cAMP–PKA pathway is associated with cAMP activating GSK-3β.

It is important to point out that only one human airway epithelial cell line and one strain of PA were used in this study. Though fluticasone propionate upregulated C/EBP β expression in BEAS-2B cells (Kimura et al., 2001), just as we found in this study, it is appropriate to extrapolate our data cautiously in other experiment conditions. Secondly, the data were derived from the *in vitro* experiments, and the *in vivo* environment is much more complex. Additional experiments with pertinent animal models will be needed to confirm this speculation.

## Conclusion

In summary, our work established a new aspect for better understanding the predilection to infections by PA with the treatment of GCs in chronic lung disease. Administrating GCs increased the interaction between PA and the host airway epithelium by upregulating the expression of SDC1. The strategies based on inhibiting SDC1 upregulation when giving GCs may provide new therapeutic approaches to correct the risk of pneumonia that occurs in administrating GCs in chronic lung disease.

## Data Availability Statement

The original contributions presented in the study are included in the article/supplementary material, further inquiries can be directed to the corresponding author/s.

## Ethics Statement

This study was approved by the Ethics Committee of Ruijin Hospital. Neither samples from patients nor animal experiments were involved in this study.

## Author Contributions

DL contributed to the experimental design, study conduct, data analysis, and manuscript preparation. Y-YZ and M-MS contributed to the experiment conduct. J-MQ contributed to the experimental design, data analysis, and revision of the manuscript. All authors had full access to all study data and had final responsibility for the decision to submit for publication. All authors have reviewed the manuscript and approved the final version for submission.

## Conflict of Interest

The authors declare that the research was conducted in the absence of any commercial or financial relationships that could be construed as a potential conflict of interest.

## Publisher’s Note

All claims expressed in this article are solely those of the authors and do not necessarily represent those of their affiliated organizations, or those of the publisher, the editors and the reviewers. Any product that may be evaluated in this article, or claim that may be made by its manufacturer, is not guaranteed or endorsed by the publisher.

## Abbreviations

DEX: dexamethasone
BUDE: budesonide
SDC1: syndecan-1
GR: glucocorticoid-receptor
ICS: inhaled corticosteroids
HSPGs: heparan sulfate proteoglycans
MOI: multiplicity of infection
GAPDH: glyceraldehyde 3-phosphate-dehydrogenase
C/EBP β: CCAAT-enhancer-binding protein β
GSK-3 β: glycogen synthase kinase 3 β
PA: *Pseudomonas aeruginosa.*
